# Editorial

**DOI:** 10.1186/1755-8166-7-S1-A1

**Published:** 2014-01-21

**Authors:** Jayesh Sheth

**Affiliations:** 1 FRIGE's Institute of Human Genetics, FRIGE House, Satellite, Ahmedabad, India

## 

The International Conference on Human Genetics and 39^th^ Annual Meeting of the Indian Society of Human Genetics organized from January 23-25, 2014 is an effort by the Foundation for Research in Genetics and Endocrinology (FRIGE), Ahmedabad and the Institute of Life Sciences, Ahmedabad University.

With the emergence of newer technologies and expansion of scientific frontiers, the scope of genetics and allied sciences has crossed several boundaries and has opened several applications in medical diagnostics and treatment strategies. Human genetics and in particular molecular genetics has demonstrated a significant contribution in prediction and detection of genetic diseases (prenatal diagnostics) with advancement of techniques like Array-CGH, FISH and many others. For instance, microarray-based comparative genomic hybridization (array CGH) is a revolutionary platform that has been developed to screen entire genome for copy number variations (CNV) and can be used as the first line investigation modality in cases of non-syndromic mental retardation, unexplained developmental delay (DD), intellectual disability (ID), autism spectrum disorders (ASD) and multiple congenital anomalies (MCA). It can also be used for molecular characterization, to size the abnormality and study the gene content etc. In the field of cancer, several genes have been identified as the target for the treatment and cure. MicroRNAs (miRNAs) is yet another exciting advancement in the genetics. It may play an important role in regulation of expression of genes involved in cell competition at the post-transcriptional level. *In silico* screening of miRNAs involved in a cell competition is an effort to identify potential miRNAs and to reduce, economize and expedite experimental work. Under these circumstances identification of few novel and functional genes in different population can play an important role to define the future strategies for the diagnosis and treatment of various diseases. Further, many studies are emerging such that nutrition and certain micronutrients such as Vitamin B_12_, Vitamin D, folic acid etc. may have influence gene expression and genetic make-up. Therefore, the theme of the conference was chosen as ‘Healthy Genes - Healthy Life’. The presentations planned and research papers received are in conformity to this theme. There is a fair mix of papers with clinical and basic topics to be presented and included in this issue of the journal. It includes a vast area from basics of genetics to the complex of array CGH, SNP arrays and Next generation sequencing; prenatal diagnosis, pre-implantation genetics, epigenomics, pharmacogenomics, in- born errors of metabolic disorders, storage disorders, latest from the human variome project, point of care medicine and nanotechnology. We feel honoured and privileged to edit this special issue of Molecular Cytogenetics that highlights the genomic science presentations during this conference.

The Indian Society of Human Genetics (ISHG) imparts knowledge related to Human genomics through annual meeting every year in January at different places of the country to encourage young students in biomedical science and invite learned scientists for the plenary talks.

FRIGE is a nationally recognised organization with national and international alliances for research in human genetics. It is involved in carrying out basic and translation research in Human Genetics and Endocrinology with a motto of services to the society and imparting knowledge to the young students and researchers.

Institute of Life Sciences is a part of the School of Science and Technology, Ahmedabad University with a motto of "Nurturing Science, Knowledge and Innovation". The vision of the Institute is to undertake world class research in nano biotechnology leading to affordable health care to enrich human and environmental health. It has already forged alliances with national and international academic organisations as well as industries.

We wish that the readers find these abstracts very interesting for their future research work.

Jayesh Sheth

Alok Dhawan

Frenny Sheth

Ramesh K. Goyal

Sanjay Singh

